# The War and Hospitals for Nervous Diseases

**Published:** 1914-11-21

**Authors:** 


### The Hospital

**The Workers' Newspaper of Administrative Medicine and Institutional Life, Administration, National Insurance and Health.**

No. 1483, Vol. LVII. SATURDAY, NOVEMBER 21, 1914. PRICE ONE PENNY.

### THE WAR AND HOSPITALS FOR NERVOUS DISEASES.

The time has now come when we may well take stock of our equipment for restoring to health those of our brave officers and men who have succumbed to the enormous strain on brain and nerves entailed by modern warfare. Already numerous cases of neurasthenia and mental breakdown have occurred, and, unless more systematic provision be made for these and others who certainly will follow, the result will be that a large proportion will drift into chronic invalidism. On the other hand it cannot be doubted that at the present day scientific methods of nerve treatment can do a great deal for this class of sufferers, and, given a reasonable opportunity, can unquestionably save many of these threatened minds. The crux of the problem before us lies in the decision as to whether there exists already adequate and suitable accommodation for the patients in view, or whether it is in the public interest that some additional means of helping them be organised. Considering the financial burdens already thrust upon the country, there must naturally be considerable hesitation before we set about the organisation of any new type of institutions which are to be supported by voluntary charity, though, as we have already pointed out, there was certainly a gap in our preparations in this respect.

Still, the work to be done in this direction is so important, meaning as it does the saving of young, strong brains of the sort which the nation needs badly just now, that if it be found that existing hospitals are unable to do what is required, then perforce the charitable public must be asked to support yet another voluntary institution at this juncture. In view of the fact that a scheme has already been mooted with the object of organising a new institution for the care of cases of traumatic neurosis, it will be as well to review at once the possibilities offered by established institutions. As a matter of fact, in London alone there are several hospitals which specially cater for nervous diseases, and the staffs of which comprise most of our leading neurologists. Of these, the National Hospital for the Paralysed and Epileptic accommodates about 150 in-patients. Further, this hospital has a well-equipped convalescent home at East Finchley, where in another quiet house surrounded by convenient grounds every opportunity can be given for the recuperation of nerve cases. The "National," as it is commonly known in medical circles, has, through the excellence of its work and the keen researches of its staff, become the established centre of English-speaking students, who are devoting special attention to neurological problems, and therefore its claims to take a responsible part in the care of officers and soldiers afflicted with nervous shock cannot be overlooked. Moreover, it so happens that at the present time two wards at this institution remain closed through lack of funds, so that its supporters may not unreasonably consider that moneys freshly subscribed for the care of the nervous should in part at least be devoted to the reopening of these wards, and to the fullest possible development of the resources of this institution, before they are spent on new organisations. Then, again, there is the Hospital for Epilepsy and Paralysis in Maida Vale, where twenty-five beds are available for the same purpose; whilst its equipment could be considerably augmented by the establishment of a convalescent branch such as could be temporarily formed without undue expense, with the loan of a substantial country house properly equipped and selected. The accommodation of these two institutions might be further supplemented by the twenty-four beds offered to the War Office by the West End Hospital for Diseases of the Nervous System in Welbeck Street. Further, it is well to remember that those overtaken by the worst psychoses—a very small proportion of the total it is to be hoped—will inevitably have to go into the mental institutions in any case.

Obviously, then, there is already accommodation for a considerable number of patients suffering from shock, and if some provision be made for convalescence the special nerve hospitals and mental institutions may be able to be of great assistance in connection with the war. There can be no doubt, however, that the chief factor in the cure of this class of patient will be a prolonged convalescence, so that it is not so much hospital accommodation that will be required as ample opportunities to place them in restful country surroundings with suitable treatment and nursing. Preliminary treatment under the care of a specialist in hospitals will be an essential in some cases, but it is the after-care in the country that will be relied on in most instances finally to turn the scale in favour of health. One essential at the moment is that Lord Knutsford and those who are acting with him should demonstrate, by the presentation of facts, exactly the type of cases, and the estimated probable number of such cases, which they hold will actually require the establishment of a new institution for their reception and treatment. It is always a grievous wrong to multiply institutions unnecessarily, and the responsibility of such a step can never be greater than at the present moment. Hence it is necessary first of all to demonstrate beyond dispute that the two institutions suggested—namely, a quiet Home in London and one in the country—are paramount. The facts we have stated as to existing hospitals may dispose of the necessity for the first home. We feel that there is much to be said, however, in favour of the provision of a quiet home in the country.

